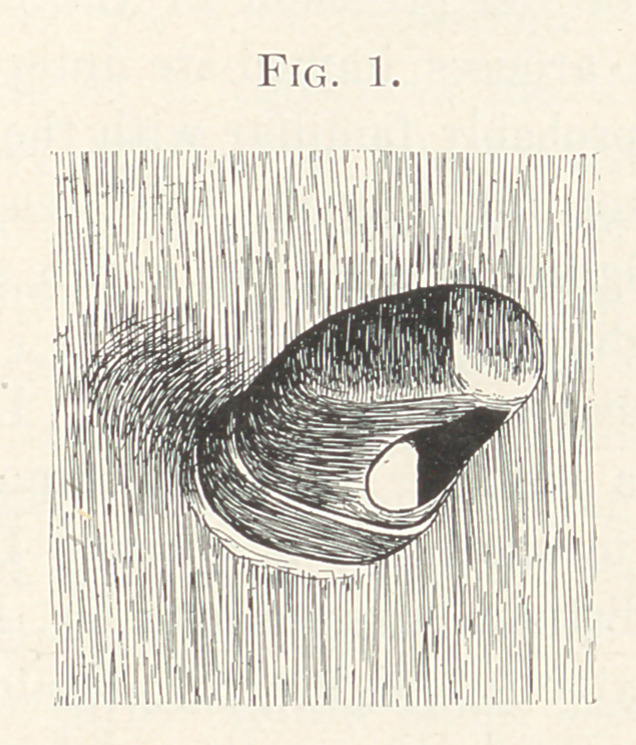# The Therapeutic Use of the X-Ray in the Oral Cavity

**Published:** 1904-05

**Authors:** George F. Eames

**Affiliations:** Boston, Mass.


### THE THERAPEUTIC USE OF THE X-RAY IN THE ORAL CAVITY.[1]

BY GEORGE F. EAMES, BOSTON, MASS.

The remarkably good results which have been recently achieved by means of the X-ray as a therapeutic agent are astonishing. The X-ray itself was accidentally discovered, as was also its chemical and therapeutic effect; its good results, therefore, are all the more surprising. The history of the discovery of the Röntgen ray, its wonderful penetrating power, and its use in photographing tissues and substances hidden from the ordinary sight are too well known to need further comment, but some attention to the nature of this wonderful electrical force, the means by which it is produced, and the methods of its application for the purpose of obtaining a therapeutic result have a claim to recognition in connection with the subject under consideration.

The X-ray is known chiefly by the phenomenon which accompanies it, as no one has yet been able to define it. Many scientists have, however, advanced theories with the object of explaining its nature and character. Many agree that it is a form of transverse

---

[1] Read before the American Medical Association, Section on Stomatology, New Orleans, May 5 to 8, 1903.

ethereal vibration, and formed in a series with sunlight and what is known as the Becquerel rays, the ethereal vibration being less in the case of ordinary light, more irregular in the Becquerel rays, and still more irregular in the X-rays.

Dr. H. P. Pratt, who has given this subject much attention, considers the X-ray as an electric current of a very high potential, which makes its circuit from the inner surface of the tube outward, perpendicularly to the surface, then radiates in straight lines until the potential falls, when the rays return to complete the circuit by the terminals. During the passage of the rays through the walls of the tube, through the atmosphere and into the body, it is accompanied by a liberation of oxygen from the body, as well as from the surrounding atmosphere. One of the most important agents in the application of the X-rays is the Crookes tube; indeed, the greater part of the technique and much of the therapeutic effect depends upon the proper handling of the tubes. The tubes themselves are usually designated as hard and soft, or, what is the same thing, high and low vacuum tubes. The condition which determines whether a tube is hard or soft is the number of molecules of residual gas in the tube; this fixes the degree of the vacuum and, consequently, the condition of the tube as to whether it is hard or soft. An X-ray tube when acted upon by the electric current has been compared to a Leyden jar: it discharges in one direction, the outer surface of the tube becomes electro-positive, and the inner surface electro-negative. The usual method of producing the Röntgen ray by means of a Crookes tube consists in furnishing electricity to it by means of a static machine,[1] or by the street current through a Rhumkorrf coil, the terminals of which are connected with a Crookes tube.

The cathodal and anodal poles are connected, the molecules of residual gas within the tube furnishing the medium through which the current is established. When the current is thus passing, these molecules of gas within the tube are driven with great force against its inner surface, and the point of contact locates the origin of the Röntgen ray.

The following ideas regarding the production of the X-ray, by

---

[1] Dr. William B. Snow advises the static machine for exciting the electric current. This should have ten revolving plates thirty to thirty-four inches in diameter. While coils are often capable of exciting high tubes, it is perilous to both coil and tubes.

Dr. H. P. Pratt, are quite pertinent to our subject. "Every molecule of gas striking the inner surface of the tube causes one or more lines of magnetic force to be thrown out at right angles to the surface of the tube. The distance to which these lines of force are projected, or, in other words, the limit of the penetrating power of the ray, depends entirely upon the potential of the tube, and this in turn depends on the force of the impact of the individual molecules of residual gas. The higher the vacuum, the less the number of molecules of residual gas in the tube; the greater the free path, the higher the potential, the greater the penetrating power. All substances through which the X-rays pass form part of the X-ray circuit. The X-ray circuit is the same as any other electrical current. It has its return forming an endless chain of molecules, arranged in series. . . . The light which is emitted from the tubes is the result of decomposition of the molecules in the atmosphere around and inside the tube. This light is not the X-ray current; the X-ray force is purely electrical and invisible.

"The softer the tube, the greater are the number of lines of force thrown out and the stronger the current which increases decomposition, but the penetrating power is decreased. We are dependent entirely upon the number of lines of force projected from the tube to bring about ionization of the tissues. Ionization means changes in the elementary structures and increase in metabolism.

"We need to have the greatest possible number of these lines of force within a given space for our best therapeutic work. This is only possible with a low or soft tube."

Having considered the nature and character of this electrical force, it becomes especially interesting to investigate the action which it has upon the various tissues of the body. Dr. Pratt suggests that "the magnetic force from the X-ray passes directly into the affected tissues. Electrolysis results, the chemical decomposition liberates oxygen, which unites with the free oxygen of the body and makes ozone. Ozone will kill every bacterium the human body possesses. The X-ray does not destroy germ life by direct action any more than does the sun's rays; the bactericidal effect of both are due to ionization, or electrolysis. Factors to be considered in X-raying are, 1, potential of the ray; 2, the resistance of the tissues to the ray; 3, the resulting intensity of the radiation. The first only is under control and is governed entirely upon conditions in the tube, which are constantly varying, but which, by corresponding changes in the current energizing the tube, the spark-gap, etc., may be made approximately constant." Experience in X-ray work has shown that for therapeutic effect, a low, or soft tube should be used, and the current increased according to the result which it is desired to obtain. The harder the tube the less the number of lines of force thrown out, and consequently the weaker the X-ray current, and the less the decomposition, but the greater the penetrating power. While it is true that the X-ray improperly or incautiously applied will certainly burn the tissues, and that the burns are very painful and serious, it is, nevertheless, of rare occurrence with the careful and experienced operator, who, being mindful of the great difference in susceptibility of patients, adapts the current, the tube, the time of exposure, and the distance of the tube from the body to the conditions he finds in his patient. He will take other precautions, such as the interposition of a celluloid screen between the patient and the tube; this prevents the germs in the air between the patient and the tube from being driven into the body. From the foregoing we may summarize the following marked characteristics of the X-ray when applied to the human body.

1. The power of penetrating deeply into the tissues.
2. Its great germicidal power.
3. The power of destroying diseased tissues with the result of new tissue being formed.

These wonderful properties of the Röntgen ray, and many others of which we know, and probably others of which we do not know, have, in actual practice, worked marvellous results, as we have ourselves seen, and as the published records have shown during the past year. It is reported that at least one hundred different diseases have yielded to the X-ray, the most notable, perhaps, being those coming under the head of malignant growths. These often occur in the mouth, and should interest the dentist. The effect of the X-ray on malignant growths is summarized by Dr. Morton as follows:

1. Relief from excruciating pain.
2. Reduction in size of new growths.
3. The establishment of the process of repair.
4. Removal of odor if present.
5. Cessation of discharge.
6. Softening and disintegration of lymphatic nodes.

7. Disappearance of lymphatic enlargements not submitted to treatment and often quite distant.

8. Removal of the cachetic color.

9. Improvement in the general health.

10. Cure, up to date, of a certain number of malignant growths.

The changes above enumerated are further described by Dr. M. F. Wheatland, who suggests that the X-ray vibrations acting on the cancer cells tend to stimulate many to maturity, at the same time breaking down the weaker ones, which are absorbed by the lymphatics and enter the circulation, producing the autointoxication so frequently observed, the number of cells reaching maturity and those undergoing destruction depending upon the intensity of the reaction established. At the same time changes take place in the small blood-vessels, their coats become thickened, narrowing their caliber, thereby reducing their blood-supply and aiding the return of the circulation to the normal.

Regarding the application of the X-ray to the mouth, the possibilities of its therapeutic effect may have a wide range. Already it has shown a marked influence over neuralgia and in the control of hemorrhage.

In the various forms of benign and malignant growths, provided they can be reached by the X-ray, we may expect the same good results that have been attained in other parts of the body. It is reported, and it has been my experience also, that the beneficent results of the X-ray are not confined alone to the part to which application is made, but that remote parts of the body also come within the range of its influence; indeed, it is often remarked by the patients that their general condition is improved, and that they have a feeling of well-being after an application of the X-ray. The report that there is an increased discharge of uric acid during this treatment seems, from the examinations which I have made, to be true. It is my belief that the application of the Röntgen ray may be effectual in the treatment of that stubborn and obscure condition generally termed pyorrhœa alveolaris, but of this I am not yet ready to report.

I am indebted to Dr. George R. Southwick, of Boston, for the privilege of reporting the following case:

F. H. B., aged forty-five, was troubled, about six months ago, with pain in the left side of the upper jaw, which was located in some of the teeth of that side. Some attention was given the teeth, and as the pain was not then constant or severe, further attention was then delayed. The pain in the jaw continuing, the patient sought advice from his physician, who suggested that he was troubled with "canker," and provided him with an antiseptic wash; but the use of this failed to relieve his condition, and recently, on account of the severity of the pain and looseness of the teeth on the affected side, he sought the advice of a surgeon, who, when he saw the case, suspected malignant trouble, and extracted the teeth on that side. He then sent the patient to Dr. Southwick, who kindly asked me to see the case and make suggestions as to an appliance for the mouth, through which the X-ray might be applied, a positive diagnosis of epithelioma having previously been made.

Finally, after several modifications, a shield was constructed which properly protected the healthy tissues and allowed the ray to reach the diseased part.

This consisted of sheet lead fourteen inches square, in the centre of which was fitted and soldered a mouth-piece which projected into the mouth as far back as the tuberosity of the jaw, closed at the end, but on the side towards the affected part a piece was cut out in order to allow the ray to pass through the opening thus made, and into the diseased tissues. (Fig. 1.) The condition

Fig. 1.

of the part at the beginning of treatment showed some loss of tissue, white patches and inflammatory conditions, and a spreading to the cheek and to the centre of the palatine vault. The treatment consisted in using a direct current of one hundred and ten volts from the street, reduced to one and a half ampères, approximately, before going to a twenty-inch coil, of a Ruhmkorff pattern. From the terminals of this coil a soft Crookes tube of twenty centimetres was used, about twelve inches from the face for about nine minutes, the face and other parts being protected by the shield.

The patient was treated in this way twice a week, and after two visits a marked improvement was shown. A further application of the X-ray was applied to the outside of the face with the object of reaching the facial nerve and controlling the neuralgia. This was effective in lessening the pain, and the good results in this direction have been progressive. The patient has, at this writing, received eight treatments, and the improvement in the mouth continues. All traces of the disease, however, have not yet been removed, and a prognosis must be withheld until a later date.

## Figures and Tables

**Fig. 1. f1:**